# Granulosis rubra nasi: A rare dermatosis not limited to nose

**DOI:** 10.1002/ski2.345

**Published:** 2024-01-31

**Authors:** Mahesh Mathur, Neha Thakur, Sunil Jaiswal, Srijana Maharjan, Anjali Shrestha, Supriya Paudel

**Affiliations:** ^1^ Department of Dermatology, Venereology, and Leprology College of Medical Sciences Teaching Hospital Bharatpur Nepal

## Abstract

Granulosis rubra nasi (GRN) is a rare genodermatosis involving the eccrine glands with an unknown aetiology. It is clinically characterized by localized hyperhidrosis, erythema, papules, pustules, and vesicles over central region of face and usually manifests during early childhood. GRN is asymptomatic, spontaneously resolves during puberty, and treatment options have inconsistent results. We hereby present a case of GRN in 38 years female with sites and dermoscopy findings not defined so far.

## Graphical abstract

Granulosis rubra nasi (GRN) is a rare genodermatosis involving the eccrine glands clinically characterized by localized hyperhidrosis, erythema, papules, pustules, and vesicles over central region of face. GRN is asymptomatic, spontaneously resolves during puberty, and treatment options have inconsistent results. We hereby present a case of GRN in 38 years female with sites and dermoscopy findings not defined so far.

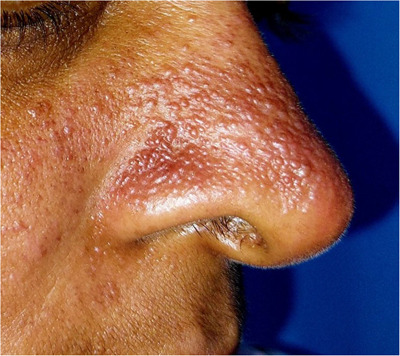

## 1 INTRODUCTION

Granulosis rubra nasi (GRN) is a benign inflammatory dermatosis involving eccrine sweat glands. GRN is also known as acne papulo‐rosacea of the nose. The first case was reported in 1901 by German dermatologist Josef Jadassohn.^1^ It has an autosomal dominant pattern of inheritance with an unknown gene locus. The aetiology of GRN is elusive but disturbance in the secretory and vasomotor function of eccrine glands leading to excessive sweat production and retention could be the probable cause. The age of onset is predominantly 7–12 years and rarely affects adolescents and adults. GRN commonly presents with hyperhidrosis and involves the eccrine glands of nose, cheeks, and chin.^2^, ^3^

## 2 CASE REPORT

**FIGURE 1:**
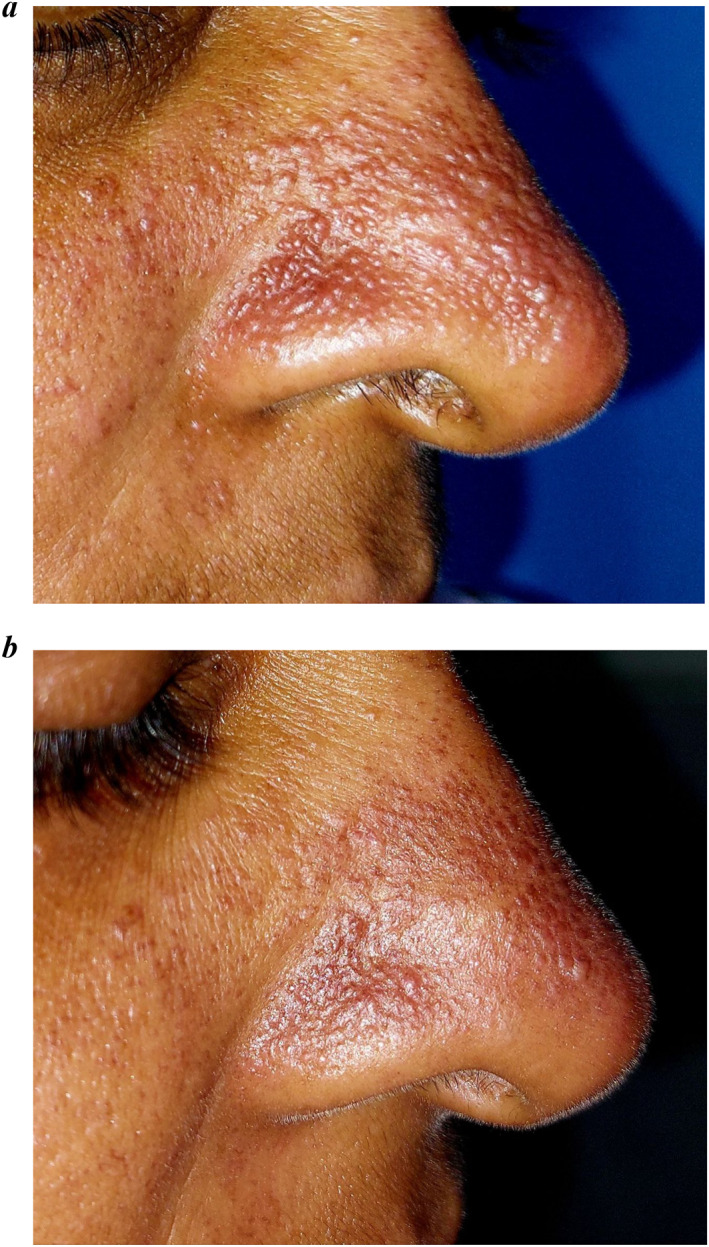
(a) Multiple erythematous and skin‐coloured papules present on dorsum and alae of nose. (b) Multiple erythematous macules with decreased number of papules on dorsum and alae of nose on follow‐up.

**FIGURE 2:**
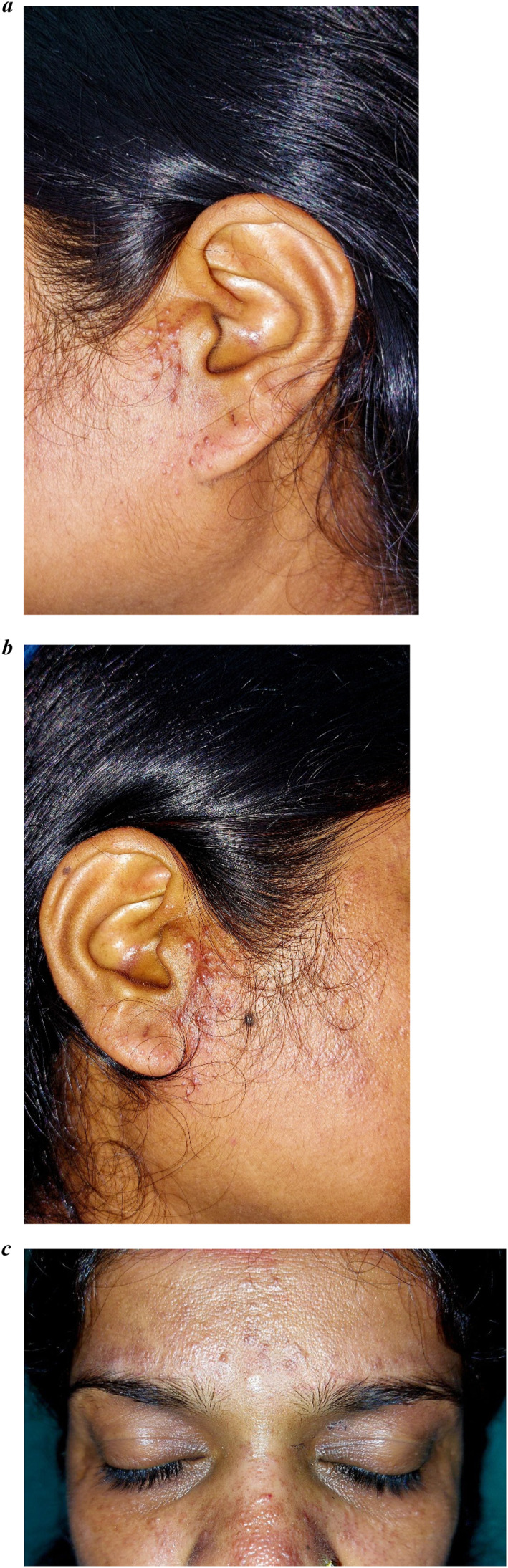
(a) Erythematous papules extending to cheeks, pre‐auricular region, and ear lobe on left side. (b) Erythematous papules extending to cheeks, pre‐auricular region, and ear lobe on right side. (c) Few erythematous papules on forehead.

A 38‐year‐old female presented with asymptomatic, multiple skin‐coloured to erythematous papules and comedo‐like lesions predominantly on dorsum, tip, and alae of nose for the last 2 years (Figure 1a). Lesions progressed to involve the forehead, infra‐orbital region, pre‐auricular area, and earlobes bilaterally (Figure 2a–c). She had history of excessive sweating on central region of face and has primary hyperhidrosis involving palms and soles. She did not have any constitutional symptoms. Her younger brother had similar lesion since childhood.

**FIGURE 3:**
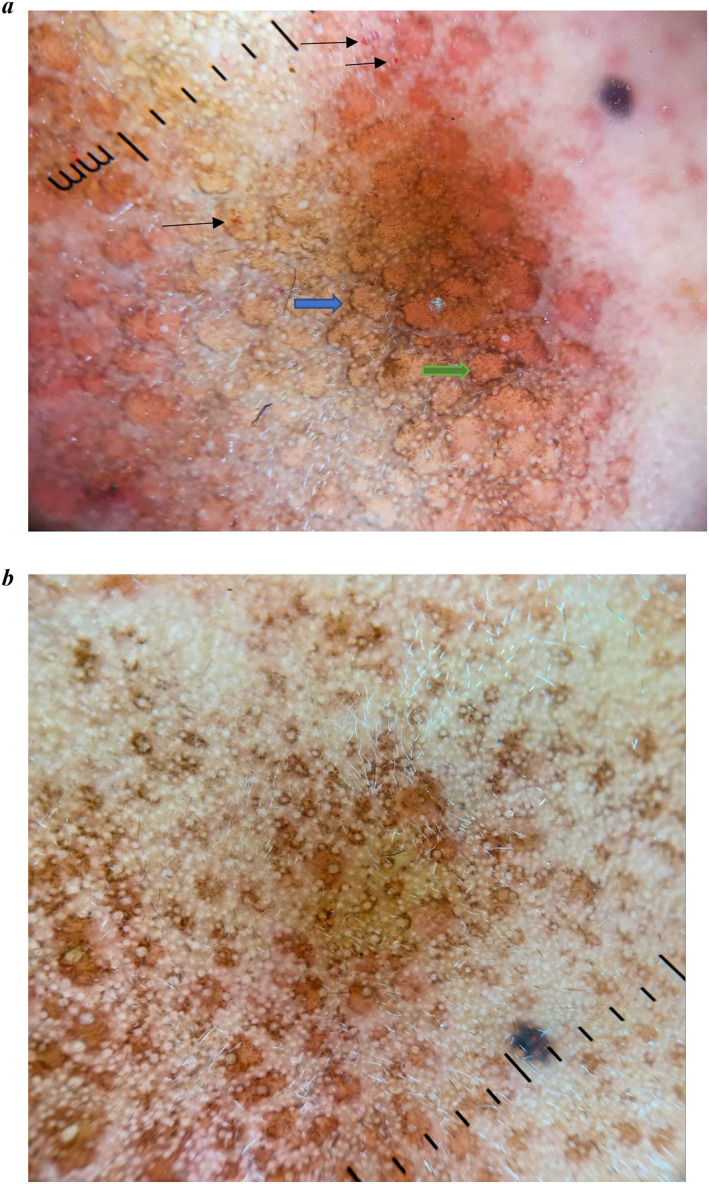
(a) Dermoscopy (DL4 polarized 10×) shows few dotted vessels (line arrow), and ill‐defined structureless areas with accentuation of pseudoreticular network (blue arrow). Grouped and scattered brown dots are seen (green arrow). (b) Dermoscopy (DL4 polarized 10×) shows decrease in size of ill‐defined structureless areas and erythema after treatment.

**FIGURE 4:**
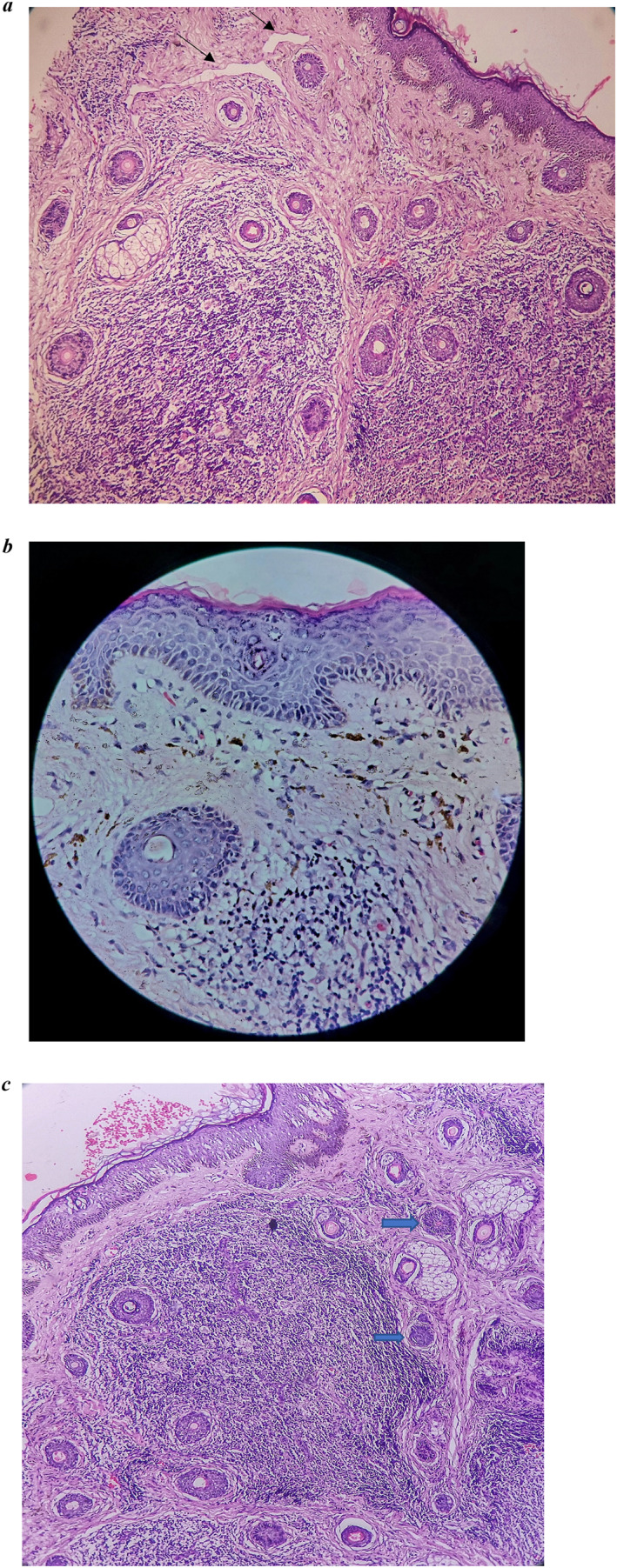
(a) Haematoxylin and eosin staining (40×) showed basal cell vacuolar degeneration, melanin incontinence, dilated lymphatics (black arrow), and blood vessels. (b) H and E demonstrate basal layer vacuolar degeneration, melanin incontinence, and inflammatory infiltrates composed of lymphocytes and histiocytes. (c) Dermal nodules composed of lymphocytes, histiocytes, and neutrophils with mononuclear cell infiltration surrounding hair follicles, eccrine sweat glands (blue arrow), and blood vessels, along with swollen endothelial cells seen in 40×.

Dermoscopy revealed brownish to erythematous ill‐defined structureless area with accentuation of pseudoreticular network pattern. Unspecified distribution of few dotted vessels seen along with grouped and scattered brown dots (Figure 3a). Biopsy was performed from pre‐auricular lesion. Haematoxylin and eosin showed basal cell vacuolar degeneration, melanin incontinence, dilated lymphatics and blood vessels (Figure 4a,b). Dermal nodule composed of lymphocytes, histiocytes and neutrophils seen. Mononuclear cell infiltration surrounding hair follicles, eccrine sweat gland, and blood vessels are noted with swollen endothelial cells. Eccrine sweat glands are enlarged (Figure 4a–c). Diagnosis of GRN was made, patient was reassured about benign nature of disease and started on topical tacrolimus 0.1% twice daily. Lesions showed significant clinical improvement and dermoscopic changes in 10 days of follow‐up (Figures 1b and 3b).

## 3 DISCUSSION

GRN is a rare focal form of hyperhidrosis that usually manifest in childhood and disappears during puberty. Primary hyperhidrosis leading to sweat retention localized to central region of face is the principal reason.^4^ GRN worsens in summer and can resolve during winter.^2^ GRN clinically manifests as asymptomatic skin‐coloured to erythematous macules, papules, pustules, and comedones‐like lesions mainly on tip of the nose. Gradually vesicles and telangiectasis involve the central face.^5^ Our patient had hyperhidrosis of palms and soles which may be a possible association or an incidental finding observed in GRN.

GRN is not associated with systemic diseases. Previous case reports in the literature revealed histopathology findings as basal cell vacuolar changes, mononuclear cell infiltration surrounding eccrine sweat ducts, dilatation of sweat glands, lymphatic vessels and dermal blood vessels with swollen endothelial cells similar to histopathology of skin lesion in our case.^5^ Dermoscopy further aids the diagnosis.

GRN is a rare condition but can mimic granulomatous rosacea, perioral dermatitis, acne vulgaris, miliaria crystallina, nevus comedonicus, and lupus erythematosus which were excluded by history, clinical presentation, morphology of skin lesion, and histopathology.^4^ GRN is treated with topical indomethacin, topical tacrolimus, oral corticosteroids, tetracycline, cryotherapy, X‐rays, and botulinum toxin A. However, lesions may reoccur despite above‐mentioned therapy. GRN has a good prognosis with minimal complication and self‐resolves in most cases.^4^

## 4 CONCLUSION

GRN being a self‐limiting and benign dermatosis, reassurance is of paramount importance. It is important to differentiate GRN from common clinical mimics. To the best of our knowledge, GRN lesions extending to sites other than the nose involving the preauricular region, earlobes, pinna, and forehead as seen in our patient have not been reported so far in the literature and our case could be the first case report of GRN with extranasal involvement. It suggests that GRN can involve any areas with eccrine sweat glands prone to occlusion and sweat retention. Adult onset as seen here is rather rare but has been described in the literature. Significant clinical response to topical tacrolimus 0.1% is seen. Topical tacrolimus was chosen considering its well‐known safety, easy availability, and effectiveness in GRN. The dermoscopy features before and after treatment are undescribed and remarkable, and histopathological changes describing almost all findings reported in literature from the past.

## FUNDING INFORMATION

This article received no specific grant from any funding agency in the public, commercial, or not‐for‐profit sectors.

## CONFLICT OF INTEREST STATEMENT

None to declare.

## AUTHOR CONTRIBUTIONS

**Mahesh Mathur**: Conceptualization (equal); data curation (equal); methodology (equal); supervision (equal); visualization (equal); writing – original draft (equal); writing – review & editing (equal). **Neha Thakur**: Conceptualization (equal); data curation (equal); investigation (equal); resources (equal); supervision (equal); visualization (equal); writing – review & editing (equal). **Sunil Jaiswal**: Investigation (equal); supervision (equal); validation (equal); visualization (equal); writing – original draft (equal). **Srijana Maharjan**: Investigation (equal); resources (equal); visualization (equal); writing – original draft (equal). **Anjali Shrestha**: Investigation (equal); resources (equal); validation (equal); visualization (equal). **Supriya Paudel**: Conceptualization (equal); formal analysis (equal); investigation (equal); resources (equal); visualization (equal); writing – original draft (equal).

## ETHICS STATEMENT

Institutional review board College of medical sciences (IRBCOMS) has provided approval. The patient in this manuscript has given written informed consent for the use of their case details (including photographs) for publication.

## Data Availability

Research data are not shared.
